# A221 GENERALIZABILITY OF RANDOMIZED CONTROLLED TRIALS TO ROUTINE CLINICAL CARE IN ULCERATIVE COLITIS

**DOI:** 10.1093/jcag/gwae059.221

**Published:** 2025-02-10

**Authors:** T Chhibba, A Frolkis, L Stein, S Lee, K Schill, E Mitseva, A Judge, M Martin, M Martin, K Novak, C Lu, M Chan, T Shukla, C Seow, G G Kaplan, A Ananthakrishnan, R Panaccione, C Ma

**Affiliations:** Gastroenterology, University of Toronto, Toronto, ON, Canada; University of Calgary, Calgary, AB, Canada; University of Calgary, Calgary, AB, Canada; University of Calgary, Calgary, AB, Canada; University of Calgary, Calgary, AB, Canada; University of Alberta, Edmonton, AB, Canada; University of Calgary, Calgary, AB, Canada; University of Calgary, Calgary, AB, Canada; University of Calgary, Calgary, AB, Canada; University of Calgary, Calgary, AB, Canada; University of Calgary, Calgary, AB, Canada; University of Calgary, Calgary, AB, Canada; University of Calgary, Calgary, AB, Canada; University of Calgary, Calgary, AB, Canada; University of Calgary, Calgary, AB, Canada; Harvard University, Cambridge, MA; University of Calgary, Calgary, AB, Canada; University of Calgary, Calgary, AB, Canada

## Abstract

**Background:**

Historically, randomized controlled trials (RCTs) have been criticized for being poorly generalizable to patients with ulcerative colitis (UC) evaluated in routine care.

**Aims:**

We aimed to evaluate the proportion of patients with UC starting an advanced therapy who would be eligible to participate in phase 3 registrational UC RCTs.

**Methods:**

We conducted a retrospective cohort analysis of UC patients starting vedolizumab, ustekinumab, or tofacitinib at two IBD clinics at the University of Calgary. Patient charts, endoscopy reports, and laboratory results were reviewed, and compared against the inclusion and exclusion criteria from five RCTs (GEMINI-I, UNIFI, OCTAVE, ELEVATE, and LUCENT). The proportion of patients who would have been deemed eligible vs. ineligible for trial participation at the time of starting a new advanced therapy was determined.

**Results:**

A total of 125 patients with UC were included: 78 (62.4%) would have been eligible for at least one of the considered RCTs (Table 1). Trial-eligible patients were younger, less likely to be exposed to prior immunosuppressants, and had higher C-reactive protein and fecal calprotectin. The most common reason for trial ineligibility was having inadequate disease activity at baseline (Mayo endoscopy subscore <2 or absence of rectal bleeding). A significantly greater proportion of patients would have been eligible for LUCENT (44.9%) compared to GEMINI-I (24.8%), OCTAVE (35.2%), or ELEVATE (35.2%) (p<0.01 for all comparisons) (Figure 1).

**Conclusions:**

Half of patients with UC starting advanced therapy in routine care may be eligible for participation in phase 3 RCTs. Disease activity is the primary reason for trial exclusion.

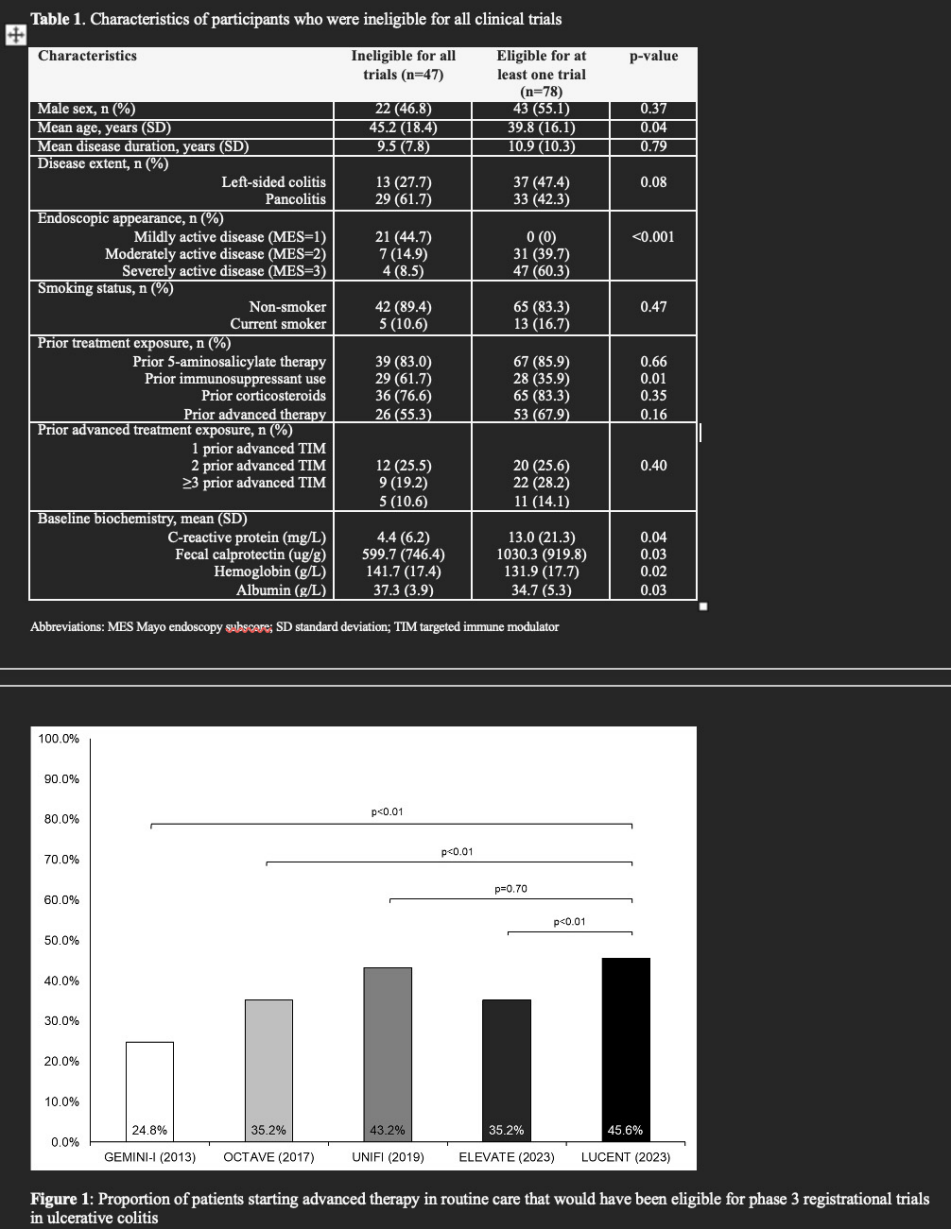

**Funding Agencies:**

None

